# Exploring Taxifolin Polymorphs: Insights on Hydrate and Anhydrous Forms

**DOI:** 10.3390/pharmaceutics13091328

**Published:** 2021-08-25

**Authors:** Fernanda Cristina Stenger Moura, Nicola Pinna, Riccardo Vivani, Gisele Elias Nunes, Aurélie Schoubben, Tania Mari Bellé Bresolin, Ivan Helmuth Bechold, Maurizio Ricci

**Affiliations:** 1 Programa de Pós Graduação em Ciências—PPGCF, Universidade do Vale do Itajaí, Itajaí 88302-202, SC, Brazil; fernandastenger@yahoo.com.br (F.C.S.M.); tbresolin@univali.br (T.M.B.B.); 2 Department of Pharmaceutical Sciences, University of Perugia, via Fabretti 48, 06123 Perugia, Italy; nicola.pinna@studenti.unipg.it (N.P.); maurizio.ricci@unipg.it (M.R.); 3 Departamento de Física, Universidade Federal de Santa Catarina—UFSC, Florianópolis 88040-900, SC, Brazil; xsele007@yahoo.com.br (G.E.N.); ivan.bechtold@ufsc.br (I.H.B.)

**Keywords:** taxifolin, polymorphism, X-ray powder diffraction, differential scanning calorimetry, thermogravimetry, equilibrium solubility, intrinsic solubility

## Abstract

Taxifolin, also known as dihydroquercetin, possesses several interesting biological properties. The purpose of the study was to identify polymorphs of taxifolin prepared using crystallization in different solvents. Data from X-ray powder diffraction, differential scanning calorimetry, and thermogravimetry enabled us to detect six different crystalline phases for taxifolin. Besides the already known fully hydrated phase, one partially hydrated phase, one monohydrated phase, two anhydrous polymorphs, and one probably solvated phase were obtained. The unit cell parameters were defined for three of them, while one anhydrous polymorph was fully structurally characterized by X-ray powder diffraction data. Scanning electron microscopy and hot stage microscopy were also employed to characterize the crystallized taxifolin powders. The hydrate and anhydrous forms showed remarkable stability in drastic storage conditions, and their solubility was deeply evaluated. The anhydrous form converted into the hydrate form during the equilibrium solubility study and taxifolin equilibrium solubility was about 1.2 mg/mL. The hydrate taxifolin intrinsic dissolution rate was 56.4 μg cm^−2^ min^−1^. Using Wood’s apparatus, it was not possible to determine the intrinsic dissolution rate of anhydrous taxifolin that is expected to solubilize more rapidly than the hydrate form. In view of its high stability, its use can be hypothesized.

## 1. Introduction

(2R,3R)-2-(3,4-dihydroxyphenyl)-3,5,7-trihydroxy-2,3-dihydrochromen-4-one, also known as taxifolin (Tax), or dihydroquercetin, is a flavonoid commonly found in conifers, such as *Larix sibirica*, *Cedrus deodara*, and *Pinus roxburghii*; however, it can be found in onions, tamarind seeds, *Milk thistle*, and other plants. In a recent study, an efficient procedure able to isolate Tax with high purity from the seeds of *Mimusops balata*, was set up [1]. Tax is the object of intense studies due to its potential application in a wide range of pharmaceutical fields. In fact, it possesses antibacterial activity, with a wide bacterial spectrum [2,3]. It was found to present hepato-, cardio-, gastro-, and neuroprotective effects. It is also a potent anti-inflammatory, analgesic, and antioxidant agent, reduces cholesterol absorption and biliary salt formation, and it was even studied for its anticancer action [1,4,5,6,7,8,9,10,11,12]. To the best of our knowledge, these interesting biological activities are strengthened by the absence of genotoxicity and oral toxicity [13]. However, as with many other flavonoids, Tax is slightly soluble in water, and this highly limits its bioavailability. Since dissolution kinetics are affected by crystalline form, it is important to investigate the presence of polymorphs obtained following Tax purification/crystallization. Over past decades, the number of papers investigating the solid state of active pharmaceutical ingredients (API) has tremendously increased, and in particular, the identification of polymorphs or pseudopolymorphs of API is gaining the interest of the scientific community [14,15,16]. Indeed, it is known that the presence of different crystalline forms can dramatically affect fundamental characteristics of the API such as chemical (reactivity), physical (shape, hygroscopicity, conductivity, density), thermodynamic (free energy, solubility, melting point, vapor pressure, and others), and mechanical (compressibility, friability, hardness, and flux) properties [17,18,19]. The determination of these features is one of the aims of the preformulation studies during drug development. Preformulation focuses on the physico-chemical properties of a new drug candidate since they influence its performance and the development of the pharmaceutical formulation.

In this study, the samples obtained by recrystallization of Tax in different solvents were characterized in order to identify their crystalline phases, composition, morphology, thermal behavior, and solubility. The results demonstrated the structural richness of Tax, for which six crystalline phases were evidenced. The solubility behavior of two of them was determined in light of their high thermodynamically stability that is of paramount importance to ensure reproducible bioavailability, safety, and efficacy profiles [20,21].

## 2. Materials and Methods

### 2.1. Materials

Tax was isolated from the seeds of the fruits of *Mimusops balata*, and its purity of 99.4% was estimated by HPLC [22,23]. This compound will be indicated as Pristine-Tax in the following. The solvents used for recrystallization of Pristine-Tax were ethanol 95% (VWR Chemicals, Milan, Italy), methanol 99.5% (Carlo Erba, Cornaredo, Italy), dichloromethane 99.5% (Panreac, Barcelona, Spain), ethyl acetate 99.5% (Sigma Aldrich, Darmstadt, Germany), chloroform 99% (J.T. Baker, Phillipsburg, KS, USA), acetonitrile HPLC grade 99.9% (Carlo Erba, Cornaredo, Italy), acetone (Carlo Erba, Cornaredo, Italy), anhydrous ethanol HPLC grade (Carlo Erba, Cornaredo, Italy), and anhydrous acetonitrile HPLC grade 99.9% (Carlo Erba, Cornaredo, Italy).

### 2.2. Crystallization

Pristine-Tax (100 mg) was dissolved in a minimum volume of each of the following solvents: acetone (AC), acetonitrile (ACN), chloroform:methanol 90:10 (CH-ME), dichloromethane:ethanol 95:5 (DM-ET), ethyl acetate (EA), ethanol (ET), and methanol (ME); these solutions were completely evaporated at 25 °C and 35% relative humidity (RH) in about 1 week under a laboratory fume hood. Tax was also dissolved in anhydrous ethanol (AET) and anhydrous acetonitrile (AACN), and these solutions were evaporated under a flux of nitrogen in about 30 min under a laboratory fume hood. In the following, the obtained samples will be indicated as Tax-solvent.

### 2.3. X-ray Powder Diffraction

When required, the samples were gently ground in an agate mortar in order to reduce crystal size, avoiding the introduction of lattice defects and strain. To minimize preferred orientations, the samples were carefully side-loaded onto a glass sample holder with about 1.5 cm × 1.5 cm dimensions and 0.5 mm deep.

X-ray powder diffraction (XRPD) data were collected in theta–theta reflection geometry with the CuKα radiation (λ = 1.54056 Å) on a D8 Advance diffractometer, equipped with a Lynxeye fast detector (Bruker AXS GmbH, Karlsruhe, Germany). The Long Fine Focus tube operated at 40 kV, 40 mA. Data were collected using an angular step size of 0.015° and a count time of 10 s per step. Diffraction patterns taken at different temperatures, in the range 25–180 °C, were collected with the help of a sample holder equipped with an electronically controlled Peltier cell. The collected patterns were handled with the help of the Diffrac.EVA software (Bruker AXS GmbH, Karlsruhe, Germany). Determination of unit cell parameters was carried out by means of an autoindexation procedure using the TREOR90 program [24]. The crystal structure of Phase 3 was solved ab initio in the real space using the EXPO software [25]. For this, a simulated annealing procedure implemented in EXPO was used. This procedure consisted of the optimization of a structural model in the real space inside the unit cell, under the symmetry operations supported by the most probable space group found. This procedure had the aim of minimizing a cost function that depended on the agreement of the experimental XRPD pattern with that calculated from the randomly generated model. The structural model was defined in terms of internal coordinates such as bond lengths, bond angles, and dihedral angles, and in this case, was only one Tax molecule, and the most probable space group found was monoclinic, *P*2_1_/*a*. The best model found with this procedure was then optimized by a Rietveld refinement procedure with the GSAS package [26,27]. The refinement involved unit cell parameters and positional atomic coordinates, background, and profile parameters, while atomic displacement factors were set to reasonable values and were not refined. Hydrogen atoms were excluded from the refinement. Soft constraints were adopted for bond lengths and angles; the statistical weight of these constraints was slowly decreased during the refinement procedure. Structural details and refined bond lengths and angles are reported in Tables S1–S5 ESI. Figure S1 shows the Rietveld plot of the last refinement cycle, while Figure S2 shows the comparison of the asymmetric unit and the packing of the anhydrous and the fully hydrated phase.

### 2.4. Scanning Electron Microscopy

Particle morphology was investigated by scanning electron microscopy (SEM) using a Field Emission SEM (LEO 1525 equipped with a GEMINI column, ZEISS, Oberkochen, Germany). Samples for analysis were prepared by depositing the powder onto an aluminum specimen stub covered with a double-sided adhesive carbon disc. The samples were then coated with chromium before imaging (100 mA, 24 s, 8 nm thickness) (Quorum Q150T ES East Grinstead, West Sussex, UK).

### 2.5. Particle Size Analysis

Mean particle size and size distribution were determined using an Accusizer C770 (PSS Inc, Santa Barbara, CA, USA) using the “single particle optical sensing” technique [28]. The powder sample (1 mg) was sonicated for 30 sec in water to allow suspension, and the results were expressed as mean volume-equivalent diameter.

### 2.6. Tapped Density Determination

Tapped density (*ρ_tapped_*) was determined with a tap density tester ERWEKA SMV 102 (Heusenstamm, Germany) following the guidelines of the European Pharmacopoeia (European Pharmacopoeia, 2008a). On the same powder sample, 10 taps, 500 taps, and 1250 taps were carried out, and the corresponding volumes (V10, V500, and V1250) were read. If the difference between V500 and V1250 was higher than 2%, 1250 additional taps were applied to the sample, and the volume was read again. This process was repeated until the difference of volume between two tapping cycles was lower than 2% (*V_tapped_*). The *ρ_tapped_* and the freely settled bulk density (*ρ_bulk_*) of powders were determined in triplicate and calculated as follows:

$$\rho_{tapped}=\frac{Powder\;mass}{V_{tapped}}$$

$$\rho_{bulk}=\frac{Powder\;mass}{V_{bulk}}$$

where *V_tapped_* and *V_bulk_* represent the tapped and bulk volume, respectively.

### 2.7. Thermal Analysis

To characterize sample thermal behavior, differential scanning calorimetry (DSC) analyses were performed using a DSC 821e (Mettler Toledo, Switzerland), equipped with a liquid nitrogen cooling system. The system was calibrated using an indium standard. Samples were sealed in aluminum pans and the covers were perforated and subjected to one heating cycle from 10 to 300 °C, at a 10 °C/min rate, under nitrogen flow (50 mL/min) against an empty pan. DSC data were treated with STARe software, and the results were expressed as the mean of three independent measurements. The samples were also submitted to thermogravimetric (TG) analysis using a Jupiter STA 490C thermal analyzer (Netzsch-Gerätebau GmbH, Selb, Germany), from 20 to 300 °C, at a 10 °C/min rate under air flow (20 mL/min). Finally, the samples were analyzed using a Mettler Toledo FP82HT Hot Stage with Olympus BH53 optical transmission microscope, equipped with an Olympus U-TV0.63XC wide zoom camera. The experiments were carried out from 30 to 250 °C at a 10 °C/min rate. The samples were placed between two microscope slides, inserted in the hot stage, and observed under a non-polarized light, with 500× magnification.

### 2.8. Infrared Spectroscopy

Attenuated total reflection Fourier transform infrared (ATR-FTIR) spectra of the samples were recorded in ATR mode using an ALPHA-P spectrometer (Bruker Optics), equipped with a Platinum ATR module that includes a single reflection 45° diamond crystal and a pressure applicator. Spectra were collected at room temperature averaging over 30 scans at a resolution of 2 cm^−1^, after determination of the background spectral intensity of the empty ATR plate. All the spectra were corrected, applying the standard “atmospheric compensation” and baseline routines implemented in the OPUS 7.5 program.

### 2.9. Stability Study

Tax-EA, Tax-AACN, and Tax-ET were stored at 40 °C and 75% relative humidity (RH). Phase conversion was monitored using XRPD, recording the diffractogram after 14 days, 1 month, and 3 months.

### 2.10. Solubility Studies

Tax-ET and Tax-EA solubility was deeply investigated, determining the equilibrium solubility with the shake-flask method, the intrinsic dissolution rate, and the dissolution profile.

#### 2.10.1. Shake-Flask Method

The equilibrium solubility of Tax was determined at 37 °C using both pH 1.2 and pH 4.5 media. Solution at pH 1.2 was prepared dissolving 2.52 g NaCl in 900 mL of ultrapure water and adjusting pH with HCl (2N); the solution was then diluted to 1000 mL with ultrapure water. Solution at pH 4.5 was prepared dissolving 2.99 g sodium acetate in 900 mL of ultrapure water and adjusting pH with acetic acid (2N); the solution was then diluted to 1000 mL with ultrapure water [29]. Briefly, an excess amount of Tax (25 mg) was added to 5 mL dissolution media, and the sample was agitated in an orbital shaker incubator (Gallenkamp, London, UK) at 150 rpm for 6 h. Samples were left for equilibrium for additional 18 h, and aliquots were sampled at 24, 48, and 72 h timepoints [30]. Centrifugation (2000 rpm, 10 min) was used to obtain a clear supernatant that was immediately diluted 1:2 (*v*:*v*) with fresh media and analyzed by HPLC to quantify Tax. Tax powder left in the centrifugation tube was suspended in fresh media and added back to the flask to replace the volume sampled. After the last timepoint (72 h), the remaining solid was collected, dried, and analyzed using XRPD and DSC. All equilibrium solubility experiments were performed in triplicate.

HPLC quantification was carried out using a chromatograph (UV/Vis 104 A, Portlab, Sulmona, Italy) equipped with a Synergi Phenomenex™ C18 (150 × 4.6 mm, 4 µm) column (thermostated at 50 °C). The isocratic elution was performed at a flow rate of 0.6 mL/min using acetonitrile:acidified water (pH 3.0, orthophosphoric acid) (80:20, *v*:*v*) as mobile phase. Each run lasted 15 min. The regression curve obtained in the concentration range 1–100 μg/mL was characterized by good linearity (*r*^2^ = 0.9992).

#### 2.10.2. Intrinsic Dissolution Rate

Tax intrinsic dissolution rate (*IDR*) was determined using the rotating disk of Wood’s apparatus [31]. Disks were obtained, compacting about 200 mg Tax with a hydraulic press at 1.2 tons for 90 s. Disks with a surface area of 0.5024 cm^2^ were introduced in the dissolution vessels of the 708 DS Dissolution Apparatus (Agilent, Carpinteria, CA, USA) containing 200 mL of the solution at pH 4.5 (same composition as for the shake-flask method) at 37 °C. The rotating disk was introduced at 3.8 cm from the bottom of the pre-filled vessel, and agitation was set at 250 rpm. Samples were withdrawn at 2.5, 5, 10, 15, 20, 30, 45, 60, 90, and 120 min, and fresh media was used to replace the volume sampled. Tax quantification in the sample was performed using HPLC, as previously described, after suitable sample dilution. Intrinsic dissolution rate experiments were performed in triplicate.

The Tax concentration was plotted over time, and the slope of the linear regression was used to calculate the *IDR* using the following equation:

$$IDR=V*k\;\frac{1}{A}$$

where *k* is the slope of the linear regression curve (µg min^−1^ mL^−1^), *V* is the dissolution medium volume (mL), and *A* is the disk surface area (cm^2^).

#### 2.10.3. Tax Dissolution Profile

Tax dissolution profile in pH 4.5 solution (same composition as for the shake-flask method) at 37 °C was determined over a 120 min period. In particular, one sample for each timepoint (2.5, 5, 10, 15, 20, 30, 45, 60, 90, and 120 min) was prepared, adding about 3 mg Tax in 1 mL pH 4.5 solution. Samples were agitated in an orbital shaker incubator (Gallenkamp, UK) at 100 rpm for the whole study duration. At the determined timepoints, samples were withdrawn, filtered with 0.45 μm pore size syringe filter (regenerated cellulose, Test Scientific, Perugia, Italy), and analyzed using HPLC to quantify Tax.

### 2.11. Statistical Analysis

Equilibrium solubility data obtained at 72 h were analyzed using a one-way ANOVA test. Results were considered significant for *p* < 0.05.

## 3. Results and Discussion

### 3.1. X-ray Powder Diffraction and Thermal Analysis

**Scheme 1:**
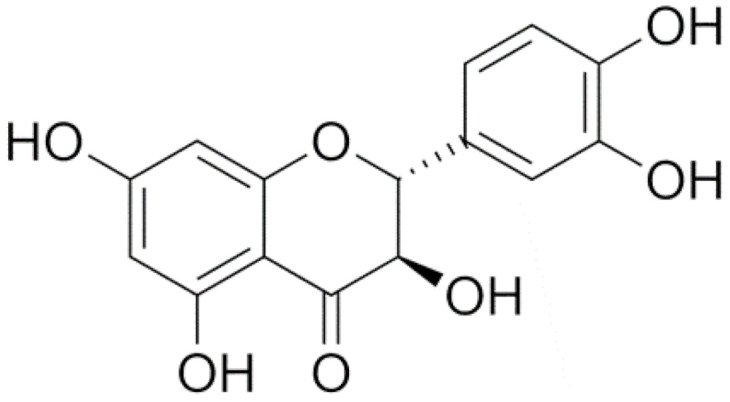
Molecular structure of Tax.

Scheme 1 shows the molecular structure of Tax.

Pristine-Tax was recrystallized using nine different solvents selected among those generally used in the pharmaceutical industry during the manufacturing of APIs and for isolation and purification of Tax. Crystallization from a single or a mixture of solvents was chosen as the method to obtain polymorphs since it does not require specific instrumentation, is simple to carry out, and is commonly used to obtain polymorphs [32,33].

**Figure 1:**
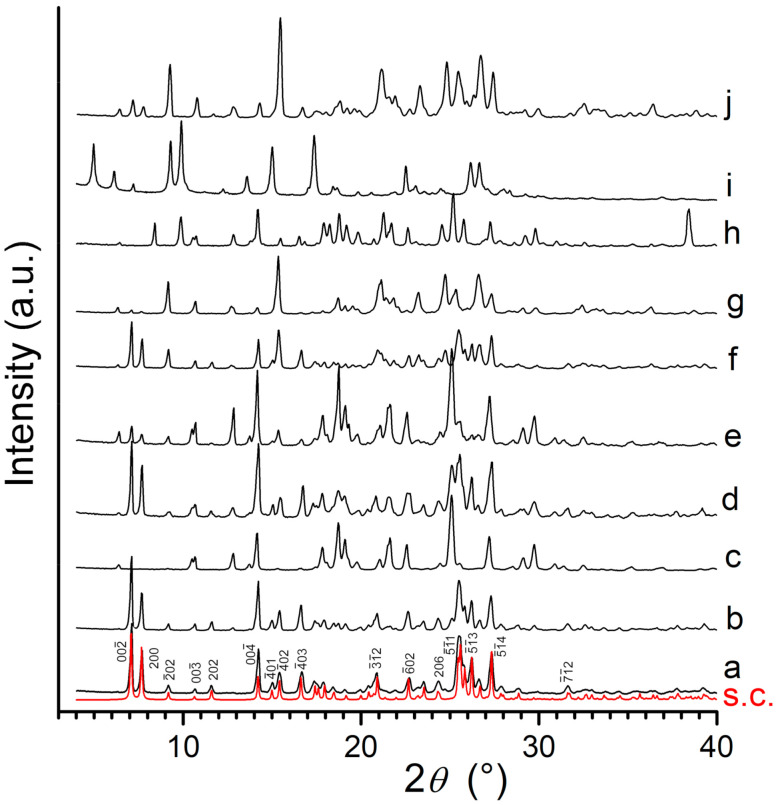
XRPD patterns of Tax-ET (**a**); Tax-ME (**b**); Tax-EA (**c**); Tax-AC (**d**); Tax-CH-ME (**e**); Tax-DM-ET (**f**); Tax-ACN (**g**); Tax-AET (**h**); Tax-AACN (**i**); Pristine-Tax (**j**). The pattern of Phase 1 calculated from single crystal structure [34] is also reported for comparison (red line, s.c.).

Figure 1 shows the XRPD patterns of the different samples prepared compared with that of Pristine-Tax.

Six different crystalline phases have been globally detected in the above samples, assessing the structural richness of this system. In some cases, it was possible to associate a unique unit cell with a defined pattern or set of diffraction peaks. In other cases, the determination of unit cell parameters failed, for several reasons, such as lack of crystallinity, the presence of more than one crystal phase, the complexity of the system, and so on. In the following, a survey of the different crystal phases identified throughout the various samples is presented.

Tax-ET and Tax-ME show the same pattern (Figure 1a,b, respectively), which corresponds to that calculated from the single crystal structure of Tax·2.5H_2_O (monoclinic, space group *C*2, *a* = 23.557(6) Å, *b* = 5.206(2) Å, *c* = 25.512(7) Å, *β* = 103.18(2)°, *V* = 3046.3(1) Å^3^) [34] (Figure 1s.c.). This fully hydrated phase will be referred to as Phase 1 in the following.

**Figure 2:**
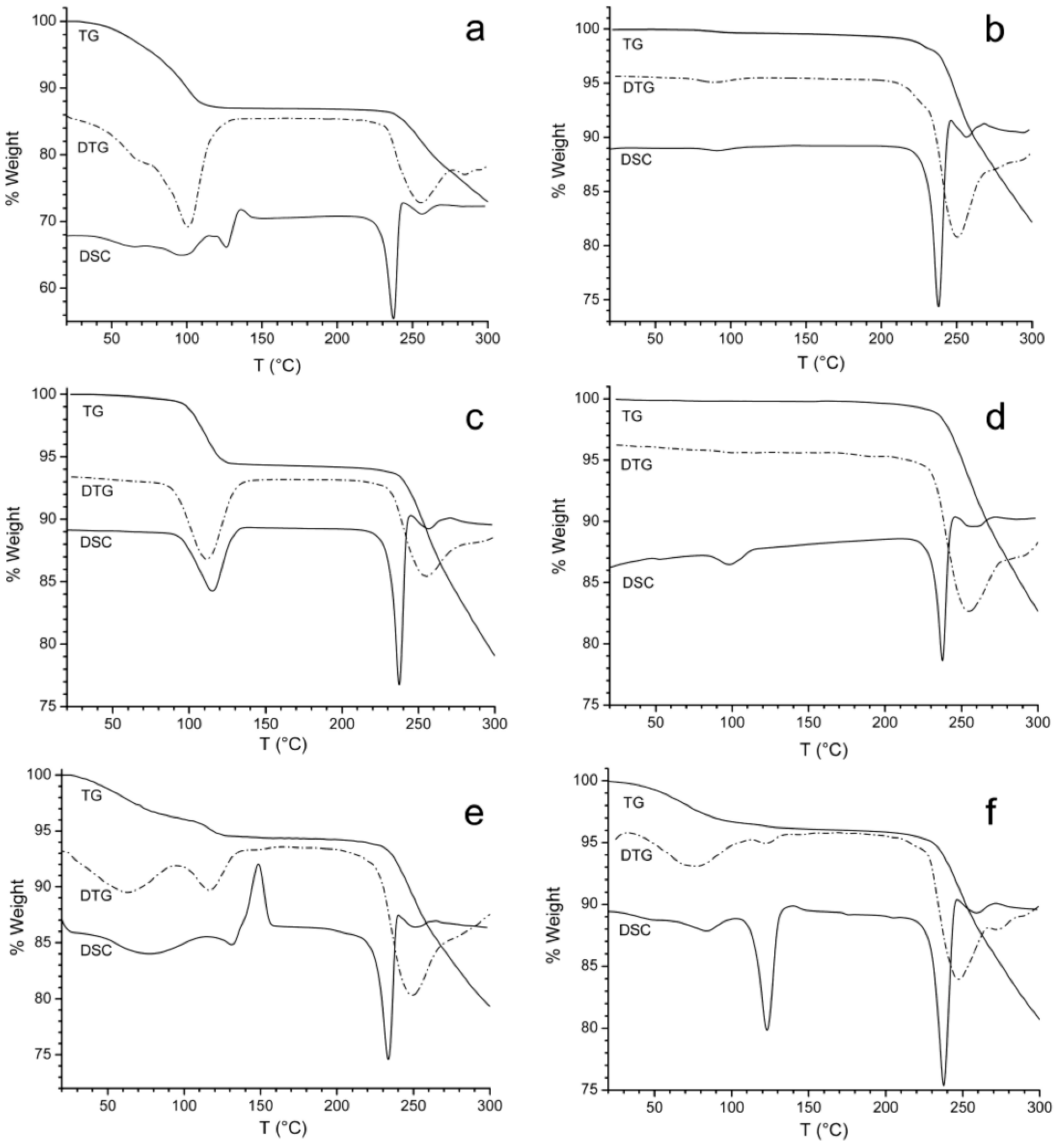
TG, DTG, and DSC curves of Tax-ET (**a**); Tax-EA (**b**); Tax-ACN (**c**); Tax-AET (**d**); Tax-AACN (**e**); Pristine-Tax (**f**).

**Figure 3:**
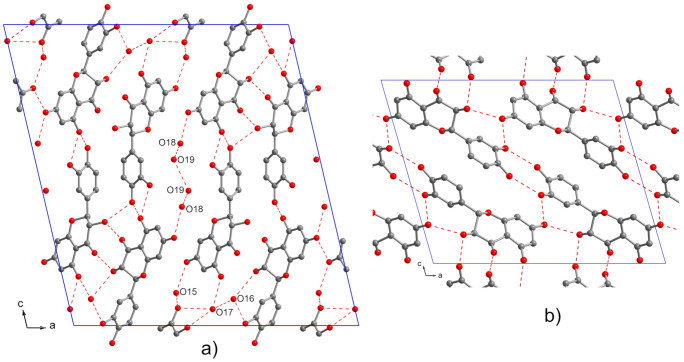
Scheme of the structure of Phase 1 (**a**); and Phase 3 (**b**). Carbon atoms are black, oxygen atoms are red, while hydrogen bonds are represented as dashed lines. Water molecules are labeled as in [34].

Weight loss at 150 °C of Tax-ET (Figure 2a) indicates the presence of 2.5 mol of water per mol of Tax, as expected. The weight loss starts at very low temperatures, before 50 °C, indicating that a part of hydration water is weakly bound to the structure. Weight loss continues with two unresolved steps in the 20–100 °C range, as revealed by the first derivative of the TG (DTG) curve and by the DSC profile that shows two partially overlapping endothermic effects, with a maximum at 65.5 °C (*T_onset_* = 42.2 °C) and 98.4 °C (*T_onset_* = 85.8 °C), respectively, and are clearly related to two distinct dehydration processes. In agreement with this behavior, the crystal structure shows that the five water molecules contained in each unit cell are not crystallographically nor chemically equivalent (Figure 3a): O18 and O19 are placed inside large channels, and O19 does not interact via hydrogen bonds with Tax, but only with O18. On the contrary, the other three water molecules, O15, O16, and O17, are closely connected to Tax molecules with an H-bond network [34].

This behavior may suggest the existence of an intermediate, partially hydrated phase between 50 and 100 °C.

**Figure 4:**
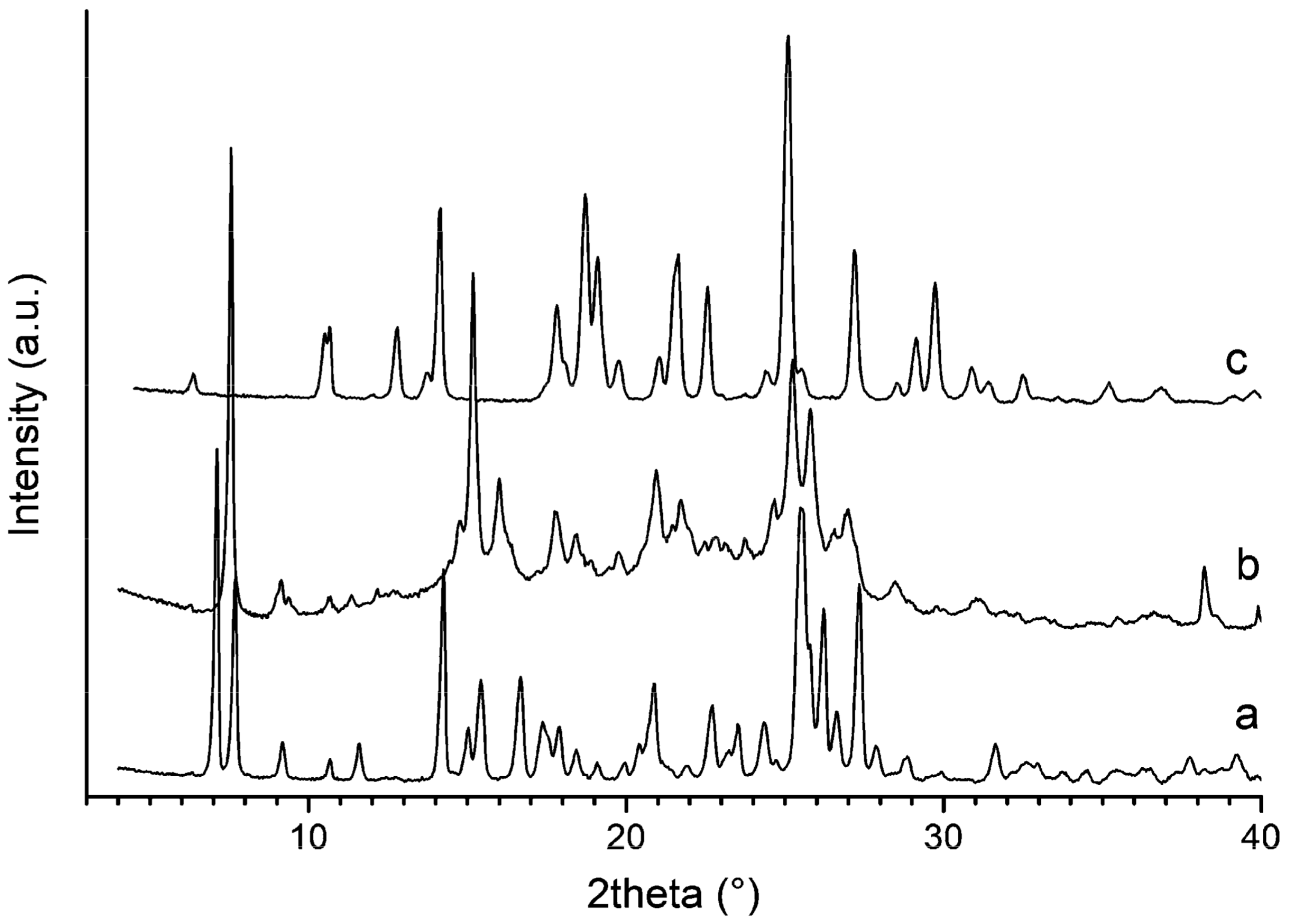
XRPD patterns of Tax-ET at 25 °C, Phase 1 (**a**); at 70 °C, Phase 2 (**b**); and at 120 °C, Phase 3 (**c**).

In agreement with TG-DSC, XRPD patterns of Tax-ET (or Tax-ME) taken at different temperatures (Figure 4) show the complete conversion of Phase 1 into a new partially hydrated Phase 2 in the 60–70 °C temperature range (Figure 4b), and a further phase transition around 100–120 °C that leads to an anhydrous Phase 3 (Figure 4c). Just after the end of the water loss, the DSC curve shows a sharp endothermic peak (*T_onset_* = 121.0 °C; *T_max_* = 126.8 °C) followed by an exothermic peak at 136.0 °C (Figure 2a) probably associated with the melt–crystallization of Phase 2 → Phase 3 transition, observed by XRPD analysis.

Phase 3 remains stable up to its melting point (DSC *T_onset_* = 230.0 °C; *T_max_* = 237.5 °C).

Phase 2 contains from 0.5 to 1.5 mol of water per mol of Tax, depending on treatment temperature and duration. At room temperature, it is stable for some hours, and tends to completely rehydrate and reconvert into Phase 1 in about 1 day.

On the contrary, Phase 3, once formed, is stable, and does not tend to rehydrate in a short time (see below).

Phase 3 has the following monoclinic unit cell: *a* = 17.4802(9) Å, *b* = 5.3121(2) Å, *c* = 14.3378(7) Å, *β* = 106.014(3)°, *V* = 1279.7(1) Å^3^ and space group *P*2_1_/*a*, with four Tax molecules per unit cell. Its structure is schematically shown in Figure 3b, where it is compared with that of the hydrate Phase 1. Molecular conformation is very close to that of Phase 1 (See Figure S2), but, due to the presence of only one Tax molecule in the asymmetric unit, the molecule packing is simpler than the hydrate form, and is characterized by the presence of two main intermolecular interactions, O1⋯O6 (d = 2.79(2) Å) and O1⋯O7 (d = 2.77(1) Å) hydrogen bonds. Other weaker hydrogen bonds are present: O4⋯O7 (d = 2.98(2) Å), O5⋯O6 (d = 3.05(2) Å), and O3⋯O3 (d = 3.15(2) Å). However, the arrangement of molecules seems to avoid the formation of efficient π–π interactions between the aromatic rings.

The XRPD pattern of Tax-EA (Figure 1c) corresponds to that of the anhydrous Phase 3, and TG and DSC curves of Tax-EA (Figure 2b) confirm that this sample is anhydrous and does not contain other solvation molecules. The weak endothermic event observed around 100 °C is probably associated with a negligible fraction of adsorbed and non-structured water. As expected, the melting point corresponds to that observed for Phase 3 (DSC *T_onset_* = 230.5 °C; *T_max_* = 237.8 °C)

Tax-AC, Tax-CH-ME, and Tax-DM-ET show a pattern that contains both Phase 1 and Phase 3 in different amounts (Figure 1d–f, respectively).

These samples, after heating at 120 °C, assume the same pattern characteristic of the anhydrous Phase 3.

The pattern of Tax-ACN (Figure 1g) shows some similarities with those of Phase 1 and Phase 3. However, after a detailed comparison of the patterns, it was possible to conclude that Tax-ACN is a different phase, dwill be indicated as Phase 4. At present, we could not find a robust unit cell consistent with the observed pattern. Weight loss of Tax-ACN (Figure 2c) around 100 °C occurs in only one step, as also assessed by DTG curve, and is associated with only one broad endothermic event in the DSC curve (DSC *T_onset_* = 95.0 °C; *T_max_* =115.5 °C). This weight loss corresponds exactly to one mol of water per mol of Tax. Phase 4 completely converts into Phase 3 upon heating at 150 °C. Additionally, in this case, its melting coincides with that of Phase 3 (DSC *T_onset_* = 230.2 °C; *T_max_* = 237.5 °C)

Due to the high stability of the anhydrous Phase 3 at 75% RH, the formation of hydrated phases of Tax-ET, Tax-ME, Tax-AC, Tax-CH-ME, Tax-DM-ET, and Tax-ACN samples very likely occurs during the crystallization process (i.e., solvent evaporation), thanks to the presence of water in the surrounding atmosphere and in the solvents used, and not for rehydration subsequent to the crystallization. Moreover, when anhydrous ethanol (AET) or acetonitrile (AACN) were used and the crystallization procedure was carried out under nitrogen, the obtained samples, Tax-AET and Tax-AACN, showed the presence of new different phases. For both of them, a high evaporation speed was adopted, which reduced the crystallization time to about 30 min, a much shorter time than that used with the non-anhydrous solvents (about 1 week). In fact, also the evaporation rate will affect crystallization, and consequently, the polymorph obtained [32].

The pattern of Tax-AET (Figure 1h) contains two sets of peaks, those characteristics of Phase 3 that can be easily recognized and subtracted, and another set of peaks not corresponding to previously observed phases in this system (Figure S3). This set of peaks is consistent with the following triclinic unit cell: *a* = 11.67Å, *b* = 10.07 Å, *c* = 7.722 Å, *α* = 100.26°, *β* = 93.25°, *γ* = 64.59°, *V* = 805.7 Å^3^, *M*(18) = 18 (see Table S6). The value of the volume of this unit cell strongly suggests that it contains only two Tax molecules. This further anhydrous phase will be indicated as Phase 5. In spite of our efforts, we were not able to obtain a sample consisting of only pure Phase 5, and therefore we could not go ahead with further structural characterization. TG and DSC analyses of Tax-AET (Figure 2d) support the hypothesis deduced from XRPD since they confirm that this sample is anhydrous and does not contain other solvation molecules.

XRPD pattern of Tax-AACN (Figure 1i) is completely different from those of all the other samples of this system. Unfortunately, we could not find a unit cell with robust statistical parameters able to index the whole pattern. This is probably due to the fact that the unit cell of this phase has a quite large volume, owing to the presence of some reflections at low 2*θ* (large *d*) values, thus exceeding the resolution of the experimental setting. We will indicate this new phase as Phase 6. Tax-AACN (Figure 2e) shows a complex thermal behavior, probably due to the presence of a large fraction of amorphous phase, also evidenced by XRPD analysis, most likely originating from the high crystallization rate adopted [35]. For this reason, quantitative aspects cannot be easily related to the crystalline portion of the sample. TG curve (Figure 2e) shows a broad weight loss correlated with an endothermic event revealed by DSC and thereafter an exothermic peak at about 150 °C that can be ascribed to the crystallization of the amorphous phase.

The XRPD pattern of Pristine-Tax (Figure 1j) corresponds to that of Phase 4 (characteristic of Tax-ACN sample) and a certain amount of Phase 1. Figure S4 shows a comparison between the two patterns, where it is possible to notice that the similarity involves both peak positions and intensities. The differences between the two patterns can be easily ascribed to the contribution of the strongest peaks of Phase 1 present in Pristine-Tax. TG and DSC curves of Pristine-Tax are reported in Figure 2f. They are globally in agreement with the presence of mainly the monohydrated Phase 4 and a smaller amount of the more hydrated Phase 1. The weight loss of Pristine-Tax at 150 °C is only slightly larger than that of Tax-ACN (6.61 vs. 5.65 %), corresponding to 1.2 mol of water per mol of Tax. The additional 0.2 mol of water, with respect to the one mol present in Phase 4, is related to a corresponding amount of Phase 1. From these data, we can calculate that Pristine-Tax is approximately composed of 80% in moles of Phase 4 and 20% of Phase 1. The DSC curve (Figure 2f) confirms that, differently from Tax-ACN, water is lost in more than one step due to the presence of Phase 1 in the sample.

In summary, besides the already known phase (Phase 1), which contains 2.5 mol of water per mol of Tax, the following phases were identified: one partially hydrated phase, Phase 2, obtained by heating Phase 1 at 70 °C; two anhydrous polymorphs, Phase 3 and Phase 5 (one of which was fully structurally characterized); one monohydrated phase, Phase 4; and one probably solvated phase, Phase 6.

Besides the different thermal behaviors shown up to 150 °C, DSC curves of all samples feature an endothermic peak clearly related to the melting event (Figure 2) [36]. The melting points of all samples are very similar despite their different crystal phase at room temperature. This reasonably indicates that all initial phases are converted into the anhydrous Phase 3 before the melting temperature. The measured melting points agree with those of commercially available samples. Tax-AACN (Figure 2e) has a significantly lower melting point (*T_onset_* = 225.3 °C; *T_max_* = 233.5 °C), most likely because the crystallization process of its amorphous fraction, detected by the exothermic peak at about 150 °C in the DSC curve, produces a poor crystalline phase, with a slightly lower melting point.

For all samples, decomposition starts just after melting, as observed by TG curves (Figure 2).

### 3.2. Scanning Electron Microscopy

**Figure 5:**
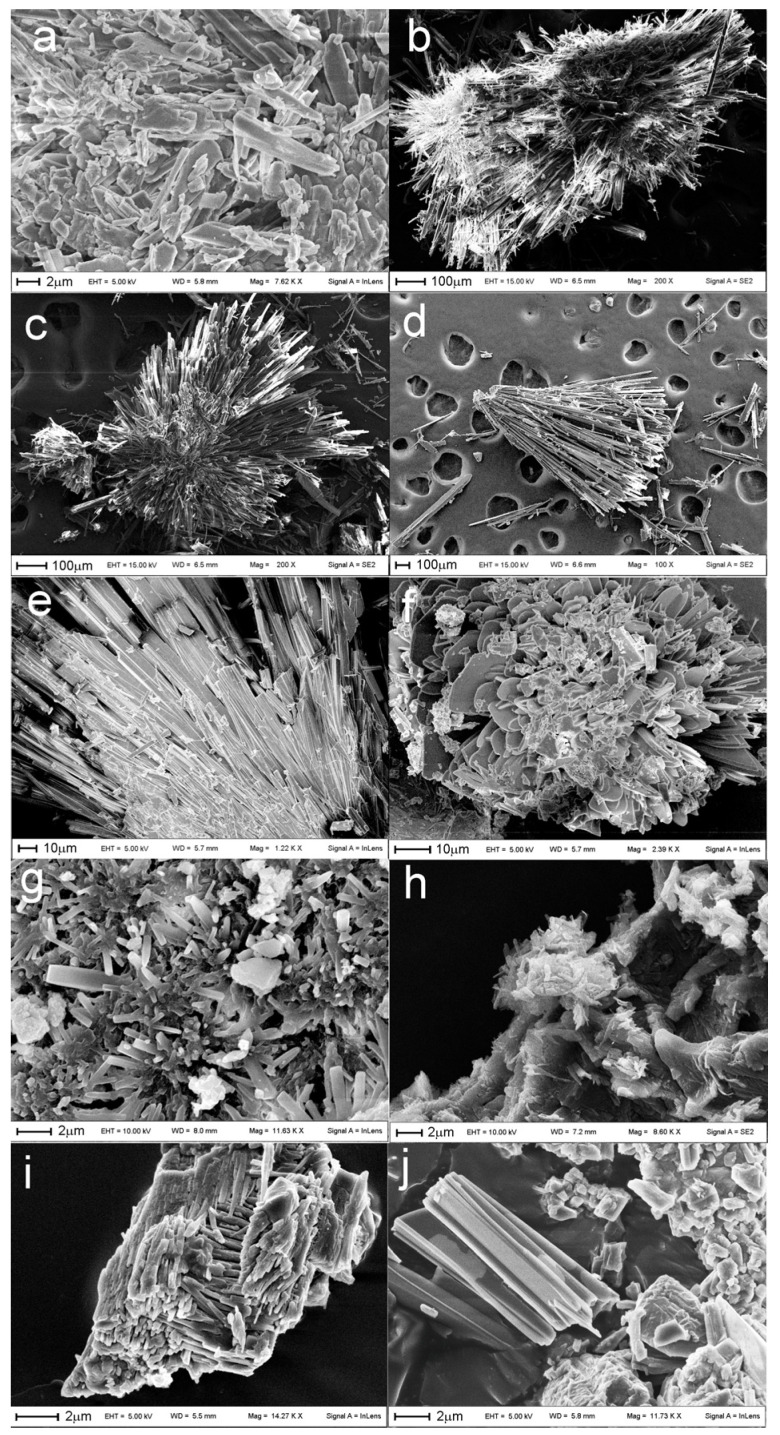
SEM images of Pristine-Tax (**a**); Tax-ET (**b**); Tax-ME (**c**); Tax-CH-ME (**d**); Tax-ACN (**e**); Tax-EA (**f**); Tax-AET (**g**); Tax-AACN (**h**); Tax-AC (**i**); Tax-DM-ET (**j**).

SEM analysis was used to investigate the influence of crystallization solvents on the shape and size of the crystals obtained (Figure 5).

Tax-ET, Tax-ME, and Tax-CH-ME (Figure 5b–d, respectively), which are characterized by similar phase composition (Phase 1), display particles characterized by needle-like shape crystals and similar size (more than 500 µm long). These crystals appear similar to those crystallized using a supercritical antisolvent process in an ethanol solution of Tax by Zu et al. [37]. Analogously, Pristine-Tax and Tax-ACN (Figure 5a,e, respectively) that have a common crystal phase (Phase 4) also display similar rod-like microcrystals, although their size is much smaller, about one order of magnitude lower for Pristine-Tax than for Tax-ACN. Microcrystals of Tax-EA and Tax-AET (Figure 5f,g, respectively) that are made of anhydrous phases tend to assume a similar platelet-like morphology. Tax-AACN (Figure 5h) consists of small prismatic crystals and a large fraction of an irregularly shaped matrix, attributed to the amorphous fraction, whose presence was evidenced by the XRPD and DSC analysis. Tax-AC and Tax-DM-ET (Figure 5i,j, respectively), which have a phase composition similar to that of Tax-ET and Tax-ME, show a different morphology, with small aggregates of prismatic crystals having dimensions around 2–10 µm.

As evidenced by the SEM analysis, according to the solvent used for its crystallization, Tax particles will have very different dimensions and shapes. It is therefore fundamental to deeply characterize Tax powders available on the market since these features will strongly affect further processing of the powder. For instance, dimensions and shape have an impact on how the powders behave in a process or as a product and can be the cause of problems faced by the industry (e.g., cohesion, adhesion, compressibility, caking, flowability) [38].

### 3.3. Infrared Spectroscopy

**Figure 6:**
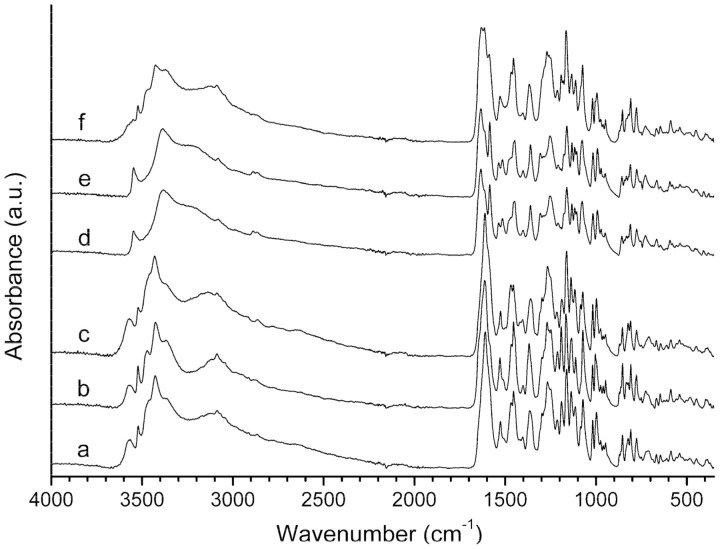
ATR-FTIR spectra of Pristine-Tax (**a**); Tax-ACN (**b**); Tax-ET (**c**); Tax-EA (**d**); Tax-AET (**e**); Tax-AACN (**f**).

The ATR-FTIR spectra of selected samples are reported in Figure 6.

The large band between 3000 and 3700 cm^−1^ is commonly ascribed to O–H stretching, while the characteristic bands at 3424 cm^−1^ and 3094 cm^−1^ correspond to the intramolecular hydrogen bonds C5–OH···O=C and C3–OH···O=C, respectively [39]. The C=O stretching of flavone was individuated at 1620 cm^−1^, while the aromatic ring vibration and, in particular, the C=C bending bands were at 1550 and 1452 cm^−1^ [40,41]. The band at 1360 cm^−1^ can be ascribed to –C–OH deformation vibration, while –C–OH stretching was found at 1142 and 1165 cm^−1^ [41]. The band at 1265 cm^−1^ can be attributed to the C–O phenol stretching [39].

By comparing the different spectra, it can be concluded that the hydration levels of samples strongly affect the FT-IR patterns. Spectra of hydrated phases, Pristine-Tax, Tax-ACN, and Tax-ET (Figure 6a–c, respectively), are very similar, as are those of the anhydrous Tax-EA and Tax-AET (Figure 6d,e, respectively) samples. The spectrum of Tax-AACN is different from other samples, probably because of its phase inhomogeneity.

### 3.4. Hot Stage Microscopy

**Figure 7:**
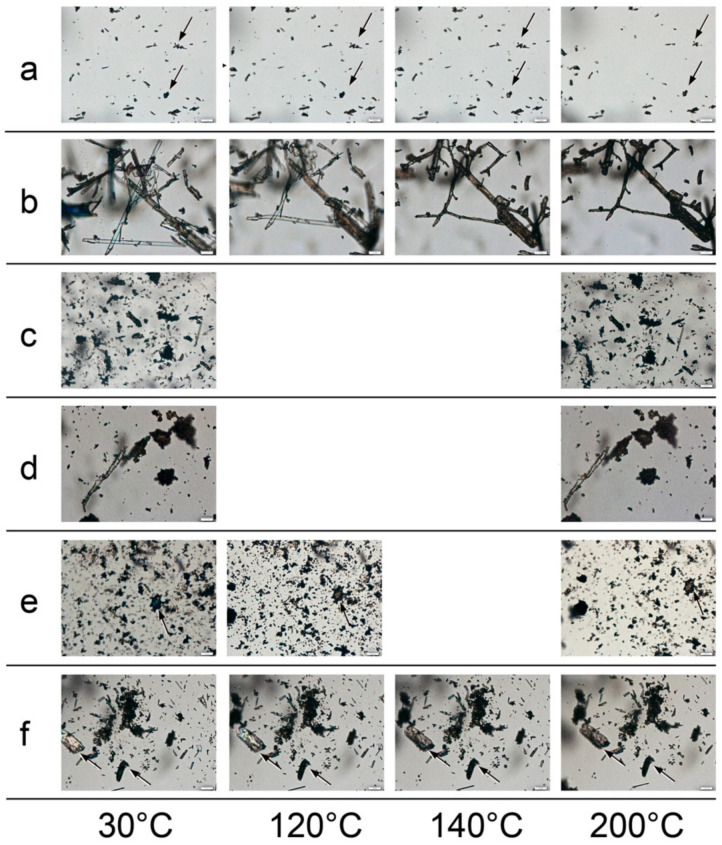
Hot stage microscopy images of Pristine-Tax (**a**); Tax-ET (**b**); Tax-AET (**c**); Tax-EA (**d**); Tax-ACN (**e**); Tax- AACN (**f**). The scale bar in the pictures corresponds to 10 μm.

With hot stage microscopy (HSM), it is possible to follow the change in morphology and light transmittance of particles during heating, and therefore to detect phase transitions and dehydration–desolvation processes [42]. HSM analyses of selected samples are reported in Figure 7 and are globally in agreement with what was observed by the other characterization techniques employed in this study.

Pristine-Tax crystals changed their color from bluish-green to brown at about 90 °C, due to the water loss, then they became opaque and darker in the range 120–130 °C, after the phase transition (arrows in Figure 7a).

Crystals of Tax-ET (Figure 7b) started to bend at about 100 °C, due to the partial dehydration and first phase transition that led to a change in the crystal lattice. After the complete dehydration and second phase transition, in the 120–142 °C range, the crystals became dark and opaque.

Tax-AET and Tax-EA (Figure 7c,d, respectively) did not change aspects during the whole analysis, in agreement with the fact that these samples are anhydrous and stable up to the melting temperature.

Tax-ACN crystals (Figure 7e) appeared bluish-green, and they became dark and opaque at about 114 °C (arrow in Figure 7e), due to the dehydration-phase transition process, in agreement with DSC and TG analysis.

Tax-AACN (Figure 7f) probably has a heterogeneous phase composition due to the presence of an amorphous fraction, so it was difficult to correlate the different analytical data. However, the dehydration (or desolvation) process occurring in the 100–140 °C range was well detected since some crystals observed became darker and opaque (arrows in Figure 7f), in agreement with DSC and TG data.

### 3.5. Stability Study

The fully hydrated Phase 1 (Tax-ET) was expected to be stable over the period investigated since water is already present in its crystalline structure. Its stability was confirmed since no modification of the diffractogram was observed after 3 months. On the contrary, Phase 6 (Tax-AACN) diffractogram was already modified 14 days after storage at 40 °C and 75% RH, indicating the high instability of its structure. Interestingly, the anhydrous Phase 3 (Tax-EA) showed to be stable over a 3-month period in drastic temperature and relative humidity conditions (Figure S5). Due to its remarkable stability, Tax-EA solubility properties were successively investigated and compared to those of Tax-ET.

### 3.6. Solubility Study

**Figure 8:**
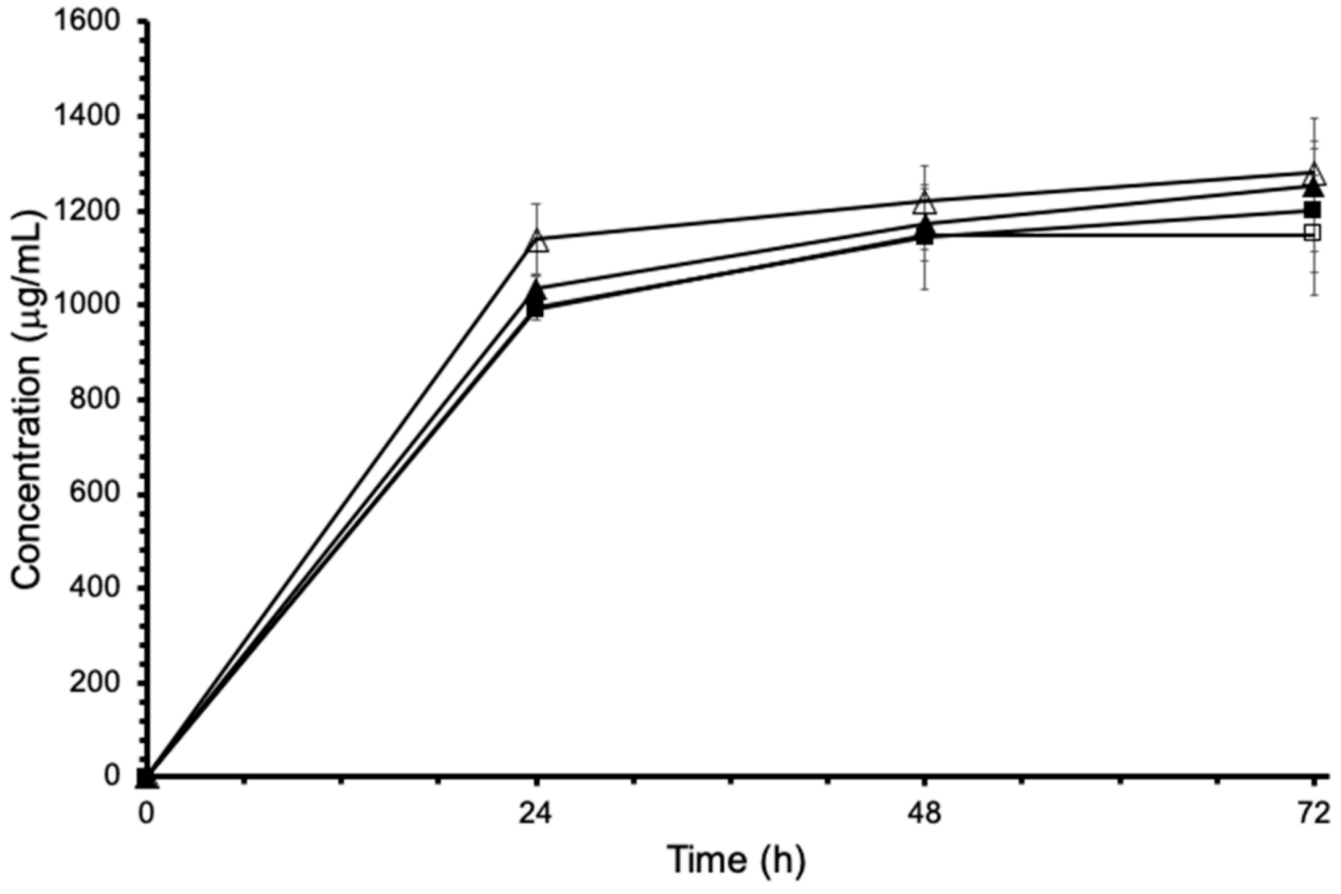
Equilibrium solubility at 37 °C of Tax-ET in pH 1.2 (■) and pH 4.5 (▲) solutions and Tax-EA in pH 1.2 (☐) and pH 4.5 (△) solutions. The scale bars correspond to the standard deviation values; experiments were performed in triplicate.

The solubility study was carried out on Phase 1 and Phase 3, which are representative of the hydrate and anhydrous forms, respectively, and showed to possess high structural stability. It is interesting to study Tax-EA solubility since the anhydrous phase could solubilize more rapidly than its respective hydrate form, as already reported for different APIs [43,44,45,46]. Equilibrium solubility was determined using the shake-flask method, considered the gold-standard method for the determination of the solubility [29,30]. Even though Tax is a non-ionizable molecule, the equilibrium solubility at 37 °C was determined in both pH 1.2 and pH 4.5 solutions, able to mimic the fasted and fed conditions. For phase separation, sedimentation is preferred to avoid filtration that can be responsible for active adsorption. Moreover, sedimentation has the advantage of not altering the dynamic equilibrium between solid dissolution and solute crystallization [30,47]. However, after sedimentation, an opalescent solution was observed, and consequently, centrifugation was employed to obtain a clear solution. As reported in Figure 8, the equilibrium solubility was almost reached after 24 h, and only a slight further increase was evidenced after 48 and 72 h. Independently of the starting Tax (i.e., Tax-ET and Tax-EA) and of the pH of the solution, Tax equilibrium solubility was comprised in the range 1.149–1.281 mg/mL. The ANOVA statistical analysis suggested that there were no significant differences between the equilibrium solubility values obtained after 72 h.

XRPD and DSC analyses (Figure S6), performed on the remaining solid recovered after 72 h, showed that there was a conversion of Phase 3 into Phase 1. On the contrary, Phase 1 did not undergo any structural modification over time. Phase 3 conversion evidenced how solubility assessment is not always an easy task. In fact, metastable polymorphs can convert progressively into a more stable form, and, in this case, the solubility value determined is that of the more stable polymorph. This was the case of sulfathiazole that possesses three polymorphs, but only one is stable during the solubility study, while the other two rapidly convert into the more stable form. In fact, already after 7 and 4 min, a decrease in the dissolution rate of polymorphs I and II was observed, respectively. Therefore, it was impossible to evaluate the solubility of the other two forms [48]. Solution-mediated transformation occurs more easily than solid-state conversion, as demonstrated from the results of the stability study. In solution, the steps leading to polymorph conversion, each one being potentially rate-limiting, are: i) dissolution of the metastable form, ii) nucleation of a more stable and less soluble form, and iii) growth of the stable crystal [49,50]. Generally, the nucleation rate is slower than the crystal growth rate of the more stable form that is slower than the dissolution of the metastable form [49,51]. Therefore, the equilibrium solubility value that has been determined is that of Tax-ET.

Tax-ET and Tax-EA *IDR* were determined using Wood’s apparatus. Unfortunately, it was not possible to determine Tax-EA *IDR* because the disk swelled and successively disaggregated after a few min from the beginning of the test. This behavior caused a variation of the surface area exposed to the dissolution media, invalidating the *IDR*. This phenomenon was already reported in the literature for diclofenac [52]. In particular, disk swelling was ascribed to the higher wettability of the anhydrous form and the faster water penetration in the tablet. The attention was then focused on Tax-ET obtaining a linear dissolution profile over 2 h (*r*^2^= 0.9937) thanks to the constant surface area exposed (Figure S7). The slope of the regression curve (*y* = 0.1417 *x* + 0.5874) (Figure S7) allowed calculating the *IDR* considering a total dissolution volume of 200 mL and a surface area of 0.5024 cm^2^. Tax-ET *IDR* was 56.4 μg cm^−2^ min^−1^, a value much lower than the non-ionizable ranitidine (47.9 mg cm^−2^ min^−1^, MW 314 g/mol) [53], but higher than non-ionizable carbamazepine (24 μg cm^−2^ min^−1^, MW 236 g/mol), griseofulvin (1.9–10.1 μg cm^−2^ min^−1^, MW 353 g/mol) [53,54], and cinnarizine (0.06–0.09 μg cm^−2^ min^−1^, MW 369 g/mol) [55]. Despite Tax possessing an equilibrium solubility that is relatively low, it will solubilize faster than other APIs already on the market.

**Figure 9:**
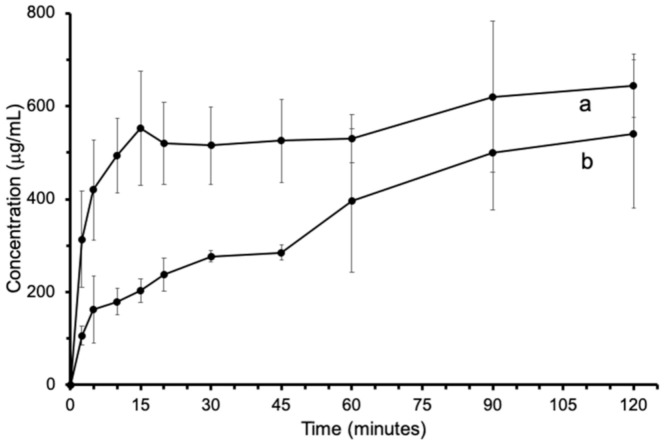
Tax-ET (**a**) and Tax-EA (**b**) dissolution profile at 37 °C in pH 4.5 solution and 100 rpm agitation. The scale bars correspond to the standard deviation values; experiments were performed in triplicate.

Lastly, Tax-ET and Tax-EA dissolution profiles were determined in pH 4.5 solution at 37 °C. Before carrying out this study, both powders were characterized in terms of density and particle mean dimensions. Phase 1 and Phase 3 showed a bulk density of 0.31 and 0.66 g/mL, respectively, while the tapped density was 0.51 and 0.82 g/mL, respectively. These values confirm the strong effect of the crystallization solvent on dimensions and shape that directly influence powder density. Particle size distributions of Tax-ET and Tax-EA evidenced the larger dimensions of Phase 1 with respect to Phase 3 particles (Figure S8) with a mean volume diameter of 29.17 and 26.96 μm, respectively. As shown in Figure 9, dissolution profiles are different and characterized by a relevant variability as evidenced by the error bars. This fluctuation can be attributed to the experimental setup that involved the preparation of a different sample for each timepoint in triplicate. Even though much attention has been paid to weight the same powder amount, particle dimensions and, therefore, the surface area can differ between samples, justifying the variability. Despite this, it is possible to highlight a faster dissolution rate during the first 10–15 min of the study and a successive decrease of the dissolution rate for both phases. Evaluating the concentration values, Tax-ET concentrations were higher than those of Tax-EA. However, this is not indicative of a higher dissolution rate because powder density, shape, and dimensions, and therefore surface area, are different, hindering a direct comparison.

In light of these solubility studies, it can be concluded that, probably, Phase 3 progressively converted into Phase 1 during dissolution, leading to an equilibrium solubility corresponding to that of Phase 1 (~1.2 mg/mL). It was not possible to determine Phase 3 *IDR* using Wood’s apparatus, and further investigation will be necessary to evidence the most suitable dissolution profile between Phase 1 and Phase 3. The use of Tax polymorphs, characterized by a better solubilization rate than Pristine-Tax, would consent to improve its efficacy and eventually extend its application field without the necessity of using excipients. In fact, reviewing the literature, several papers disclose the use of excipients as a strategy to increase Tax bioavailability [56,57,58,59,60].

## 4. Conclusions

Tax isolated from *Mimusops balata* was recrystallized in different solvents, and six different crystalline forms, among which polymorphs and solvated phases, were detected using several physico-chemical characterization techniques. For some of these forms, it was possible to determine their unit cell parameters, and for two of them, it was possible to determine their crystal structure ab initio from X-ray powder diffraction data. Interestingly, the solubility study evidenced a complete conversion of the anhydrous form into the hydrate form after 72 h. Since Tax’s anhydrous form was stable for 3 months at 40 °C and 75% RH, its use could be hypothesized. These results highlight the relevance of optimizing the procedures for the crystallization of Tax in the different phases individuated to exploit their different physico-chemical properties.

## Supplementary Materials

The following are available online at https://www.mdpi.com/article/10.3390/pharmaceutics13091328/s1, Table S1: Structural data and refinement details for Phase 3; Table S2: Atomic positional parameters for Phase 3; Table S3: Bond distances for Phase 3; Table S4: Intermolecular bond distances for Phase 3; Table S5: Bond angles for Phase 3; Table S6: TREOR90 output for the autoindexing procedure for Phase 5; Figure S1: Rietveld plot for Phase 3; Figure S2: Asymmetric unit and labelling scheme for Phase 3 (a); comparison for superpositions of a Tax molecule (b) and the whole packing in Phase 3 (grey and red) and in Phase 1 (green) (c). The overlap began with the alignment of the two rows of molecules indicated by the yellow ellipse; Figure S3: XRPD patterns of Tax-AET and Tax-EA; Figure S4: XRPD patterns of Pristine-Tax and Tax-ACN; Figure S5: XRPD patterns of Tax-EA obtained after 14 days, 1 month, and 3 months storage at 40 °C and 75% RH with respect to the starting Tax-EA; Figure S6: (A) Tax-EA XRPD patterns of the starting Phase 3 (b) and of the remaining solid recovered after 72 h of the equilibrium solubility study (a). (B) DSC curves of starting Tax-ET and Tax-EA and of the remaining solid recovered after 72 h of the equilibrium solubility study. Powder Tax-ET at pH 4.5 was not enough to perform the DSC analysis; Figure S7: Intrinsic dissolution rate of Tax-ET in pH 4.5 solution using the rotating disk apparatus and 250 rpm agitation; Figure S8: Particle cumulative size distribution of Tax-ET (blue) and Tax-EA (red).
