# Supplementary material for: Exploring Taxifolin Polymorphs: Insights on Hydrate and Anhydrous Forms
Source: Pharmaceutics. 2021 Aug 25;13(9):1328. doi: 10.3390/pharmaceutics13091328 (PMC8469002; doi:10.3390/pharmaceutics13091328)
Supplement: Supplementary file 1 [file pharmaceutics-13-01328-s001.zip › pharmaceutics-1340000.pdf]

pharmaceutics

Supplementary Materials: Exploring taxifolin polymorphs: insights on hydrate and anhydrous forms

Fernanda Cristina Stenger Moura, Nicola Pinna, Riccardo Vivani, Gisele Elias Nunes, Aurélie Schoubben, Tania Mari Bellé Bresolin [1], Ivan Helmuth Bechold [3] and Maurizio Ricci

Table S1. Structural data and refinement details for **Phase 3.**

**Empirical formula**	**$C_{15} H_{12} O_7$**
Formula weight	304.25
Crystal system	Monoclinic
Space Group	P 1 2_1/a 1 (14)
a/ Å	17.4802(9)
b/Å	5.31212(19)
c/Å	14.3378(7)
α/deg.	90
β/deg.	106.0140(27)
γ/deg.	90
Volume/Å^3	1279.70(10)
Z	4
Calculated density/g·cm^{-3}	1.579
Data range/ 2θ·deg^{-1}	4 - 80
Wavelength/Å	1.54056
N. of data points	4470
Reflections collected, unique	966
N. of parameters	114
N. of restraints	73
R_p	0.0486
R_{wp}	0.0636
R_F2	0.06106
GOF	3.38

$R_p = \Sigma \mid I_o - I_c \mid / \Sigma I_o$; $R_{wp} = [\Sigma w(I_o-I_c)^2 / \Sigma wI_o^2]^{1/2}$; $R_F2 = \Sigma \mid F_o^2 - F_c^2 \mid / \Sigma \mid F_o^2 \mid$; $GOF = [\Sigma w(I_o-I_c)^2 / (N_o - N_{var})]^{1/2}$

Table S2. Atomic positional parameters for **Phase 3.**

Atom	x/*a* (Å)	y/*b* (Å)	z/*c* (Å)
C1	0.9699(7)	0.5344(21)	0.1944(8)
C2	0.9181(8)	0.5520(27)	0.2525(10)
C3	0.8512(7)	0.8920(26)	0.1521(7)
C4	0.8990(8)	0.8590(26)	0.0922(11)
C5	0.9545(8)	0.6688(24)	0.1092(9)
C6	0.8586(9)	0.7325(25)	0.2326(13)
O1	1.0251(7)	0.3498(19)	0.2108(9)
O2	0.7990(5)	0.7104(16)	0.2764(8)
C7	0.7566(4)	0.9298(16)	0.2718(5)
C8	0.7131(5)	0.9937(16)	0.1650(5)
C9	0.7750(5)	1.0368(18)	0.1112(6)
O3	0.7740(6)	1.2257(22)	0.0597(10)
O4	0.8925(7)	1.0220(22)	0.0177(8)
C10	0.6969(5)	0.9015(17)	0.3327(6)
C11	0.7060(8)	1.0520(28)	0.4135(9)
C12	0.6525(8)	1.0350(22)	0.4684(11)
C13	0.5993(8)	0.8364(21)	0.4536(9)
C14	0.5913(6)	0.6765(17)	0.3751(7)
C15	0.6472(9)	0.6913(22)	0.3215(12)
O5	0.5653(8)	0.7727(20)	0.5242(8)
O6	0.5485(7)	0.4641(20)	0.3726(8)
O7	0.6650(6)	1.1970(21)	0.1568(8)

Table S3. Bond distances for **Phase 3.**

Atom 1	Atom 2	Length (Å)
C1	C2	1.392(6)
C1	C5	1.376(6)
C1	O1	1.351(6)
C2	C6	1.385(6)
C3	C4	1.365(6)
C3	C6	1.408(6)
C3	C9	1.509(6)
C4	C5	1.375(6)
C4	O4	1.356(6)
C6	O2	1.363(5)
C7	O2	1.373(6)
C7	C8	1.547(6)
C7	C10	1.541(5)
C8	C9	1.510(6)
C8	O7	1.354(6)
C9	O3	1.243(6)
O3	C9	1.243(6)
O4	C4	1.356(6)
C10	C11	1.381(6)
C10	C15	1.398(6)
C11	C12	1.380(6)
C12	C13	1.384(6)
C13	C14	1.386(6)
C13	O5	1.350(6)
C14	C15	1.401(6)
C14	O6	1.349(6)

Table S4. Intermolecular bond distances for **Phase 3.**

Atom 1	Atom 2	Length (Å)
O1	O6	2.79(2)
O1	O7	2.77(1)
O4	O7	2.98(2)
O5	O6	3.05(2)
O3	O3	3.15(2)

Table S5. Bond angles for Phase 3.

Atom 1	Atom 2	Atom 3	Amplitude (°)
C2	C1	C5	119.8(4)
C2	C1	O1	119.8(4)
C5	C1	O1	119.1(6)
C1	C2	C6	119.9(4)
C4	C3	C6	120.2(4)
C4	C3	C9	116.7(4)
C6	C3	C9	119.8(6)
C3	C4	C5	120.5(4)
C3	C4	O4	118.7(4)
C5	C4	O4	120.7(5)
C1	C5	C4	119.6(6)
C2	C6	C3	118.7(5)
C2	C6	O2	118.2(4)
C3	C6	O2	121.3(9)
C6	O2	C7	112.1(4)
O2	C7	C8	110.2(4)
O2	C7	C10	109.3(4)
C8	C7	C10	111.0(4)
C7	C8	C9	108.3(4)
C7	C8	O7	112.6(4)
C9	C8	O7	110.5(4)
C3	C9	C8	113.9(3)
C3	C9	O3	120.6(4)
C8	C9	O3	122.1(5)
C7	C10	C11	118.7(3)
C7	C10	C15	120.7(4)
C11	C10	C15	118.8(5)
C10	C11	C12	120.1(4)
C11	C12	C13	119.7(4)
C12	C13	C14	120.2(4)
C12	C13	O5	119.5(6)
C14	C13	O5	119.3(4)
C13	C14	C15	118.8(4)
C13	C14	O6	117.7(5)
C15	C14	O6	119.9(4)
C10	C15	C14	119.2(3)

Table S6: TREOR90 output for the autoindexing procedure for **Phase 5.**

```
  A = 11.665695  0.010584 A   Alfa  =100.263527  0.055482 Degrees
  B = 10.065780  0.012088 A   Beta  = 93.248985  0.045177 Degrees
  C =  7.722361  0.004526 A   Gamma = 64.586708  0.086142 Degrees
 Unit cell volume =     805.74 A**3
    H   K   L SST-OBS  SST-CALC   DELTA   2TH-OBS 2TH-CALC D-OBS
    1   0   0 0.005371 0.005347     0.019   8.406    8.387 10.5101
    1   1   0 0.007384 0.007376     0.005   9.859    9.854  8.9641
    0   1   0          0.007393                      9.865
    2   1   0          0.018053                     15.443
   -1   1   0 0.018110 0.018103     0.003  15.468   15.465  5.7238
```

0	1	1	0.020648	0.020649	-0.000	16.523	16.524	5.3605
2	0	0	0.021400	0.021387	0.005	16.824	16.819	5.2655
1	-1	1	0.025116	0.025079	0.013	18.237	18.224	4.8605
2	1	1	0.030618	0.030648	-0.010	20.155	20.165	4.4021
-2	0	1	0.032324	0.032328	-0.001	20.715	20.716	4.2844
0	-2	1	0.033972	0.033904	0.022	21.242	21.221	4.1792
2	2	1	0.045100	0.045075	0.007	24.522	24.515	3.6271
1	-2	1	0.049700	0.049647	0.014	25.763	25.749	3.4552
1	1	2	0.053794	0.053789	0.001	26.822	26.821	3.3211
0	1	2	0.054458	0.054466	-0.002	26.990	26.992	3.3008
-2	-1	2		0.054547			27.013	
-2	-3	1	0.057738	0.057760	-0.005	27.807	27.812	3.2057
0	-2	2	0.058766	0.058796	-0.007	28.059	28.066	3.1775
0	-3	1	0.067867	0.067895	-0.006	30.201	30.207	2.9568
2	-2	1	0.075982	0.076085	-0.022	32.001	32.024	2.7944
4	0	0		0.085548			34.014	
0	3	1	0.085737	0.085744	-0.001	34.052	34.054	2.6307
-3	1	1		0.085851			34.076	

Number of obs. Lines = 18
Number of calc. Lines = 23

M(18) = 18 AV.EPS.= 0.0000261
F(18) = 27.(0.008077, 90)

HESS weights used

M cf. J.Appl.Cryst. 1 (1968) 108
F cf. J.Appl.Cryst. 12 (1979) 60

0 lines are unindexed.

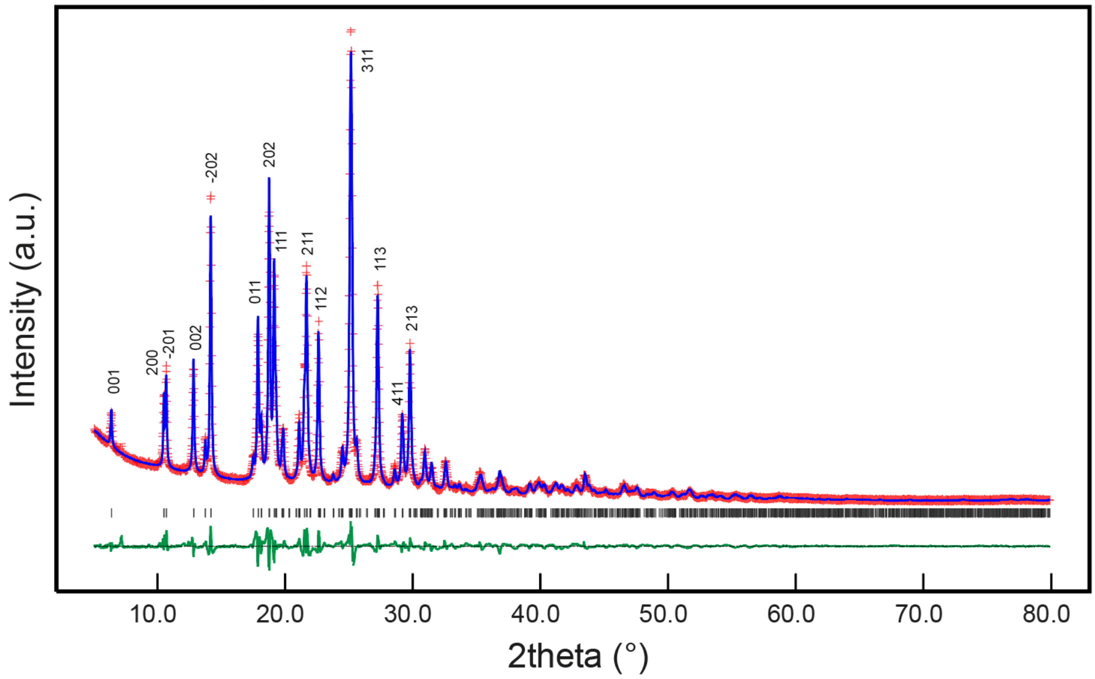

Figure S1. Rietveld plot of the last refinement cycle for **Phase 3**, showing the calculated profile (blue), the experimental pattern (red cross), and their difference (green).

Black marks indicate the calculated positions of Bragg peaks. Miller indices of most intense peaks are indicated.

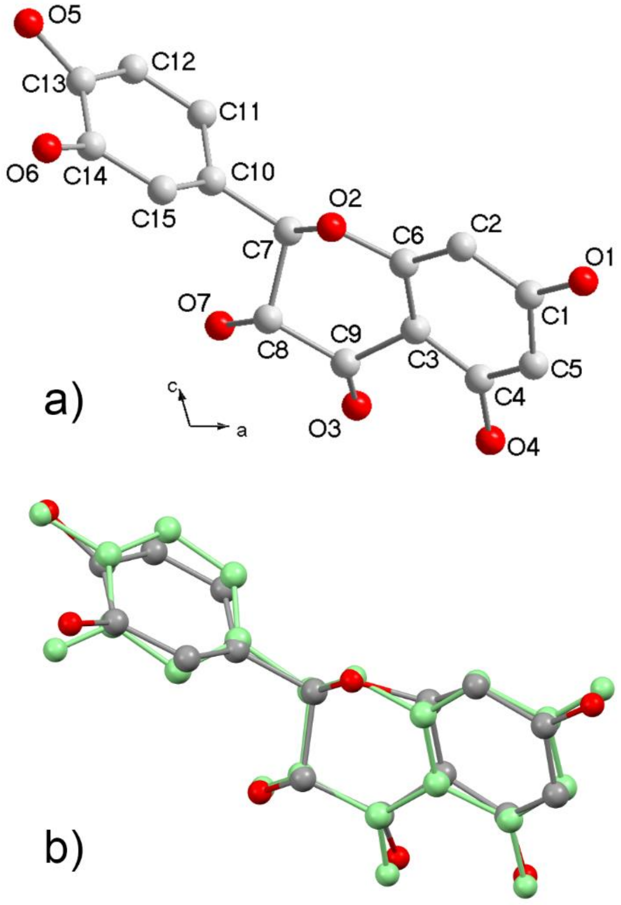

Figure S2. Asymmetric unit and labeling scheme for Phase 3 (a); comparison for superpositions of a Tax molecule in Phase 3 (grey and red) and in Phase 1 (green) (b).

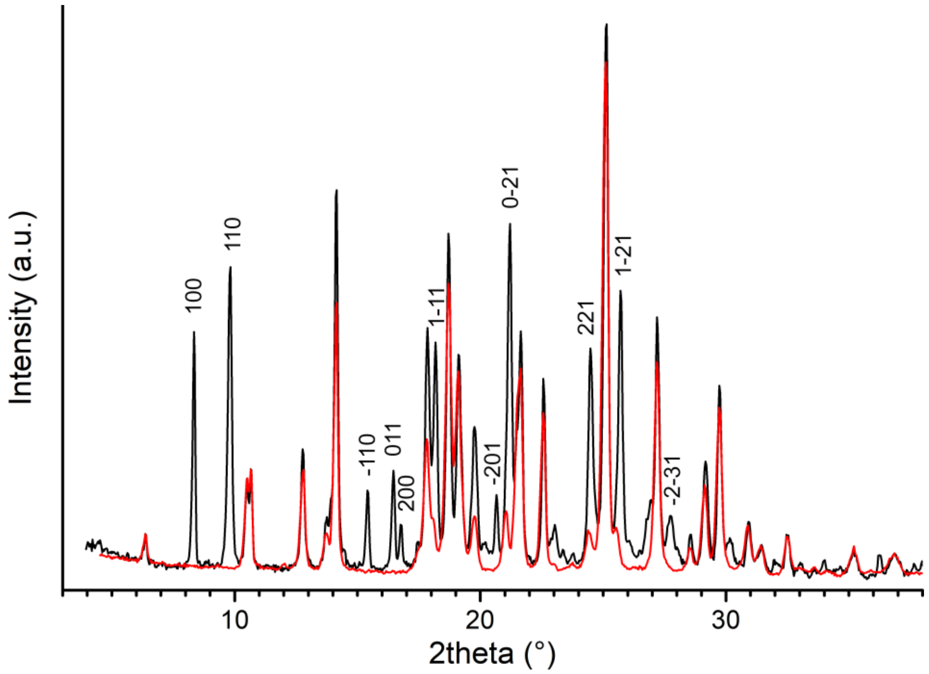

Figure S3. XRPD patterns of Tax-AET (black) and Tax-EA representative of **Phase 3** (red). Miller indices are referred to the unit cell obtained by an autoindexation procedure carried out with TREOR90 on the peaks not belonging to **Phase 3** (see Table S6) and assigned to **Phase 5**.

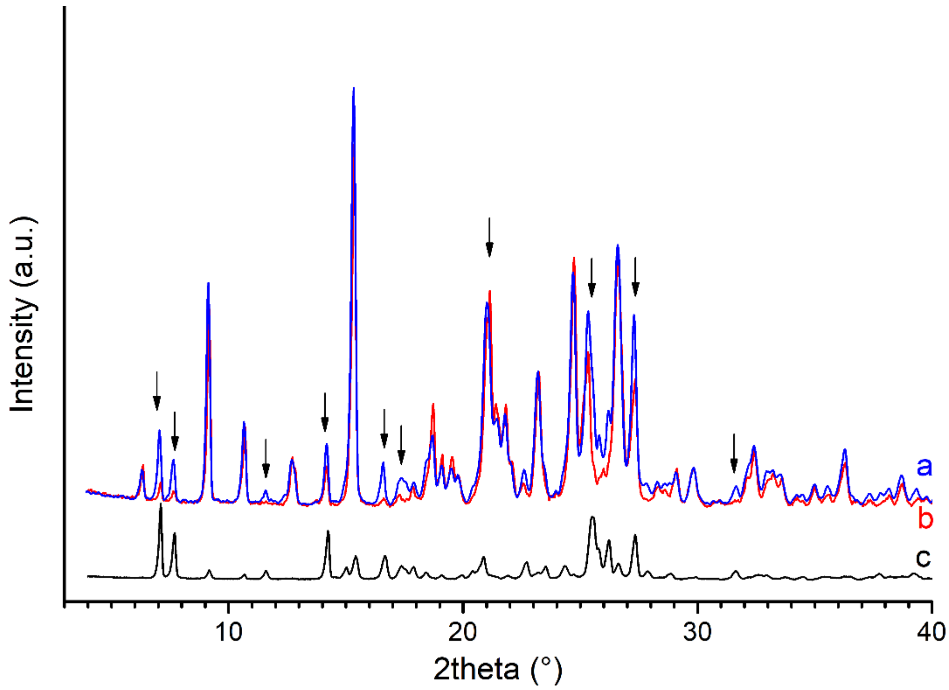

Figure S4. XRPD patterns of Pristine-Tax (a, blue line) and Tax-ACN (b, red line). The pattern of Tax-ET (**Phase 1**) is also reported, not in scale, for comparison (c, black line). Black arrows show the positions of intense peaks of Tax-ET that correspond to the regions with a relevant difference between the patterns of Pristine-Tax and Tax-ACN.

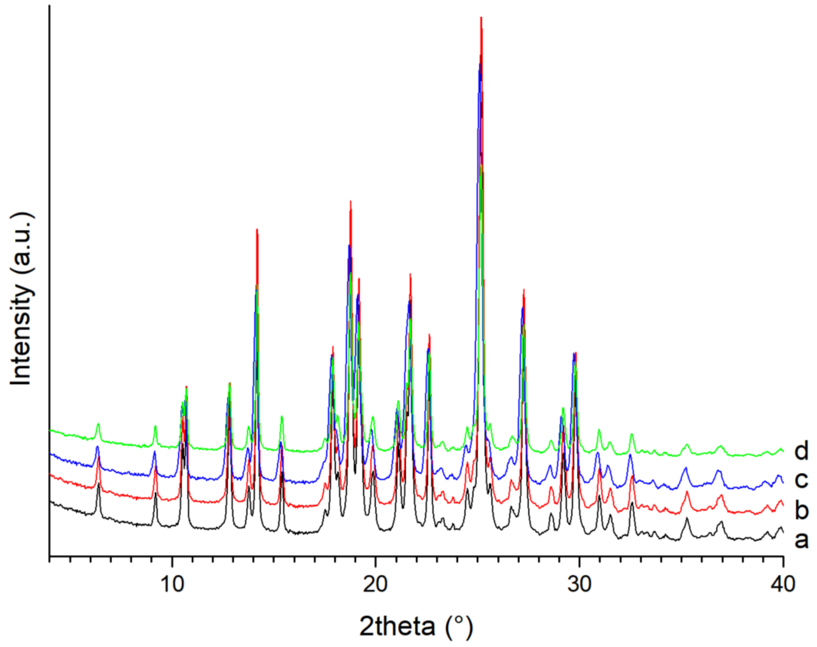

Figure S5. XRPD patterns of Tax-EA obtained after 14-day (b), 1-month (c), and 3-month (d) storage at 40 °C and 75% RH with respect to the starting Tax-EA (a).

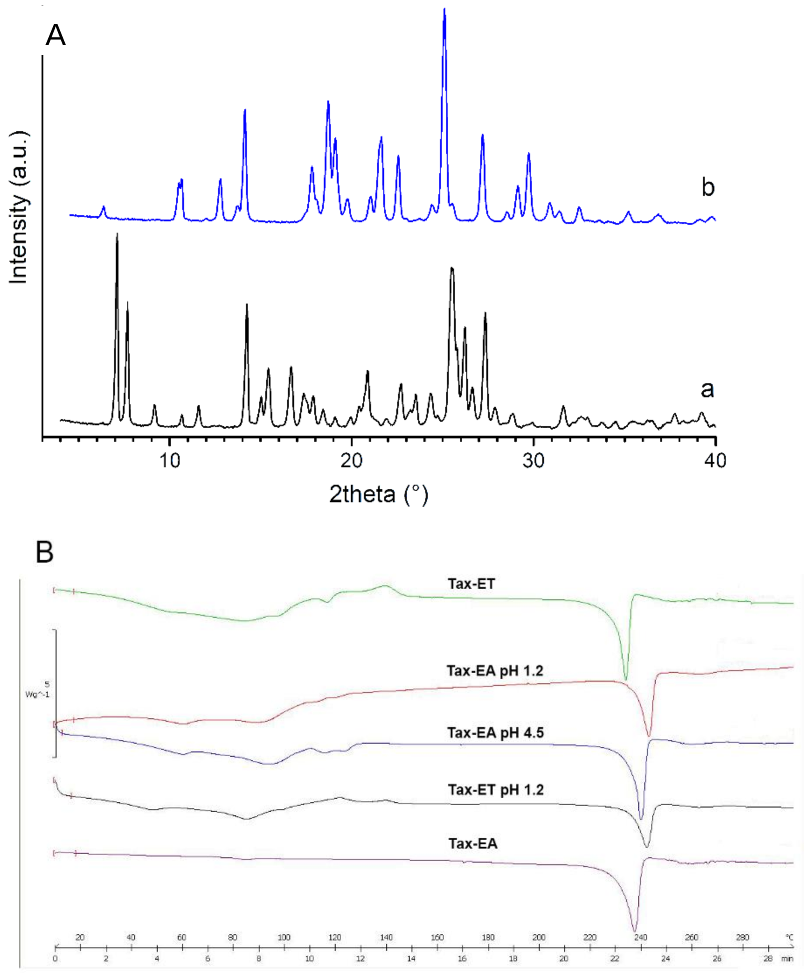

Figure S6. (A) Tax-EA XRPD patterns of the starting Phase 3 (b) and of the remaining solid recovered after 72 h of the equilibrium solubility study (a). (B) DSC curves of starting Tax-ET and Tax-EA and of the remaining solid recovered after 72 h of the equilibrium solubility study. The amount of Tax-ET powder recovered at pH 4.5 was not enough to perform the DSC analysis.

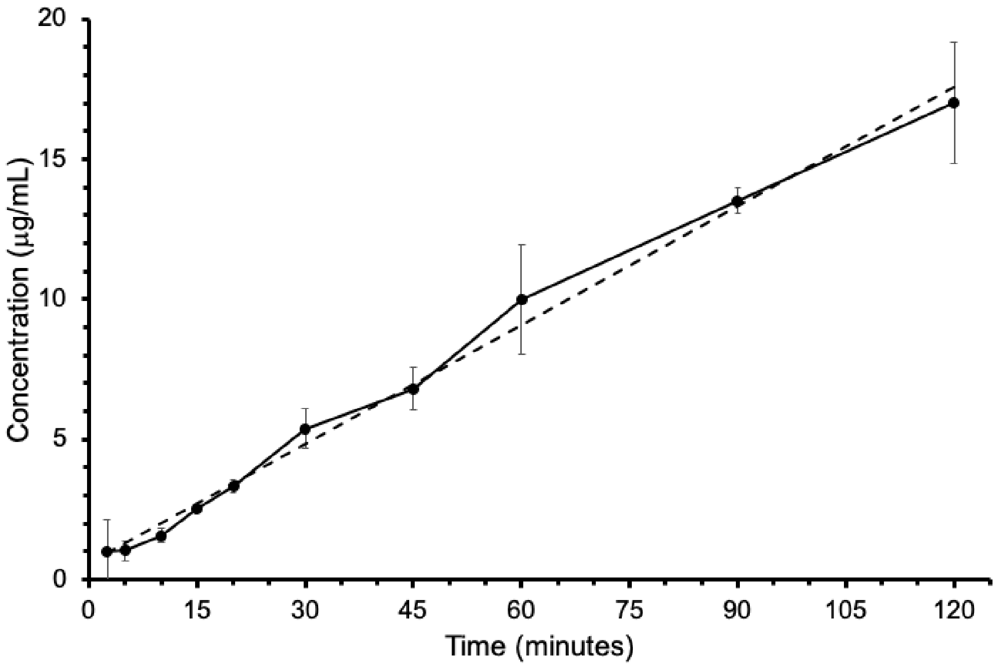

Figure S7. Intrinsic dissolution rate of Tax-ET in pH 4.5 solution using the rotating disk apparatus and 250 rpm agitation.

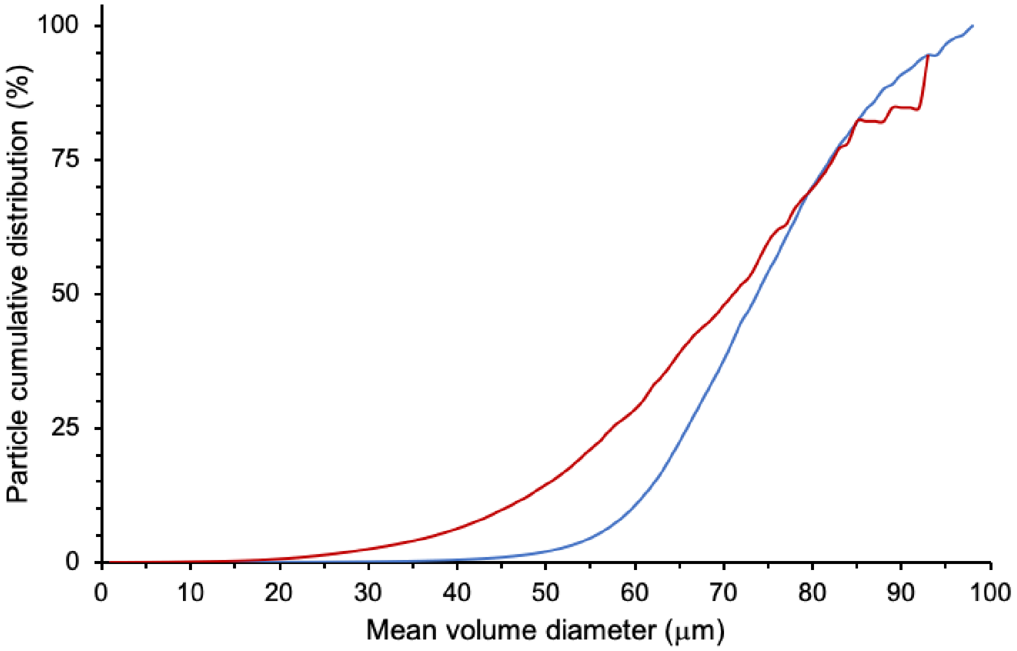

Figure S8. Particle cumulative size distribution of Tax-ET (blue) and Tax-EA (red).

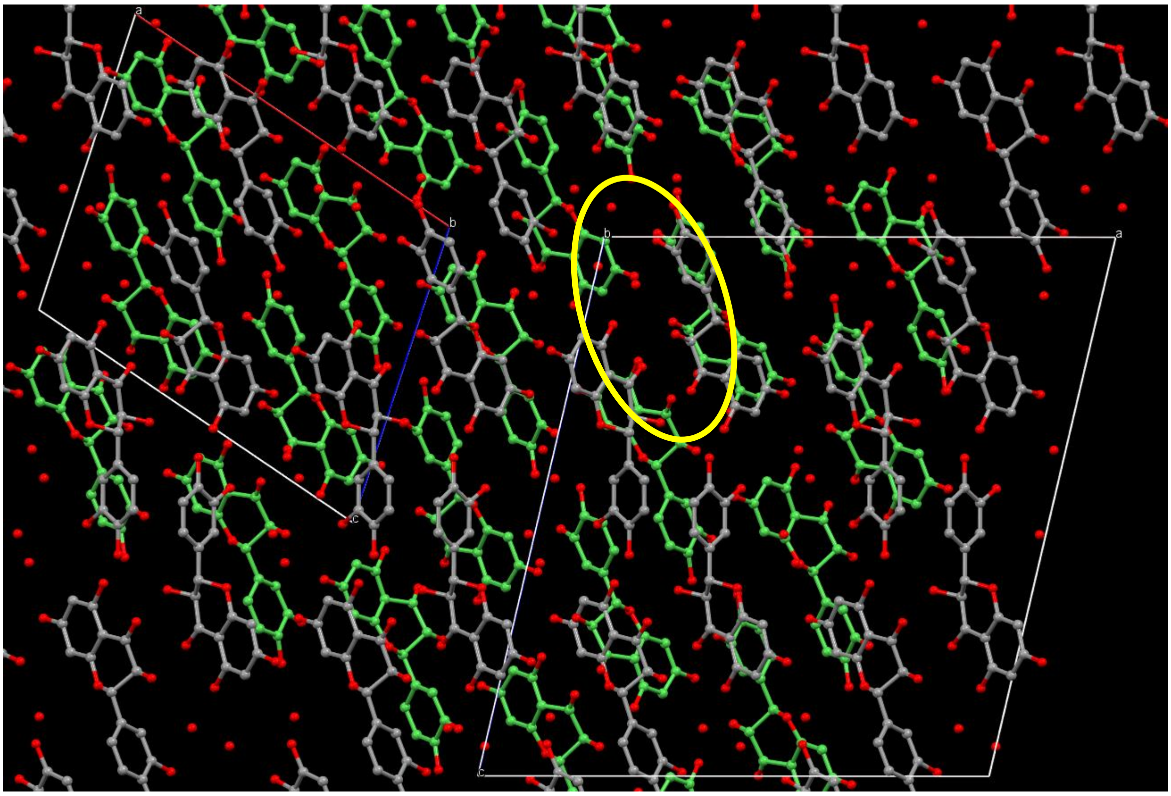

Figure S9. Superposition of Phase 3 (grey and red) and Phase 1 (green) crystal structures. The overlap began with the alignment of the row of molecules indicated by the yellow ellipse.
